# Taxonomic status and redescription of *Magneuptychia nebulosa* (Butler, 1867) (Lepidoptera, Nymphalidae, Satyrinae) with a lectotype designation

**DOI:** 10.3897/zookeys.503.9156

**Published:** 2015-05-14

**Authors:** Shinichi Nakahara, Mario Alejandro Marín, Cristóbal Ríos-Málaver

**Affiliations:** 1 McGuire Center for Lepidoptera and Biodiversity, Florida Museum of Natural History, University of Florida, Gainesville, FL 32611, USA; 2 Grupo de Investigación en Sistemática Molecular, Universidad Nacional de Colombia, Sede Medellín. Calle 59A No 63-20. Bloque 16-112 Medellín, Colombia; 3 Departamento de Biologia Animal, Instituto de Biologia, Universidade Estadual de Campinas (UNICAMP), Cidade Universitaria–Zeferino Vaz, Caixa Postal 6109, Barão Geraldo 13083-970; 4 Grupo de investigación en Ecología y Biogeografía, Universidad de Pamplona, Colombia; 5 Laboratorio de Biología de Organismos, Centro de Ecología, Instituto Venezolano de Investigaciones Científicas (IVIC), km 11 vía Panamericana Altos de Pipe, Apartado postal 20632, Caracas, 1020-A

**Keywords:** Euptychiina, Neotropical, Taxonomy, Venezuela, Morphology, Euptychiina, Neotropical, Taxonomía, Venezuela, Morfología

## Abstract

A redescription of *Magneuptychia nebulosa* (Butler, 1867), a poorly known euptychiine butterfly, is given here, and accurate distributional data are provided for the first time. Taxonomic status of this taxon has been discussed by comparing its morphology against its possible congeners. In addition, lectotype designation for *Magneuptychia nebulosa* is provided in order to objectively establish the identity of this taxon and consequently stabilize the nomenclature.

## Abstract (es)

Se presenta una redescripción de *Magneuptychia nebulosa* (Butler, 1867), una mariposa euptychina poco conocida y se brinda por primera vez datos de su distribución. Se discute el estatus taxonómico de este taxón por comparación de su morfología con la de posibles congéneres. En adición, se provee un lectotipo para *Magneuptychia nebulosa*, en búsqueda de establecer objetivamente la identidad de este taxa y consecuentemente estabilizar su nomenclatura.

## Introduction

*Magneuptychia* Forster, 1964 is one of the most diverse genera in the subtribe Euptychiina (Lepidoptera: Nymphalidae: Satyrinae: Satyrini), containing 32 described species and many undescribed species (Lamas 2004, Brévignon 2005, Brévignon and Benmesbah 2012). When Forster (1964) established the genus *Magneuptychia*, he simultaneously established the closely related genus *Argyreuptychia* Forster, 1964, which was subsequently synonymized under *Cissia* Doubleday, 1848 by Miller (1968). *Magneuptychia* is distinguished from *Cissia* by the former’s larger and more robust wingspan, its complete absence of an eyespot on the upper wing surface, and its more developed uncus (Forster 1964). However, species of both genera are poorly known, so precise character states are uncertain and not ideal for diagnoses (Forster 1964). Recent phylogenetic studies indicate that some species of *Cissia* and *Splendeuptychia* Forster, 1964 are closely related to *Magneuptychia* (Peña et al. 2010), and did not recover *Magneuptychia* as a monophyletic group, indicating that species composition between these genera must be revised thoroughly. In order to achieve this, it is necessary to reestablish the identity of those species currently placed in these genera using concrete diagnostic characters.

This paper focuses on the scarce and poorly known species *Magneuptychia nebulosa* (Butler, 1867). This specific epithet was previously found to have been misapplied to other taxa and almost no accurate information was available regarding the species’ taxonomy, biology, and distribution. Therefore, a redescription of *Magneuptychia nebulosa* based on a morphological analysis is provided, enabling future researchers to confidently identify this taxon. Two related taxa, *Magneuptychia mimas* (Godman, 1905) and *Magneuptychia alcinoe* (C. & R. Felder, 1867) were also studied in details and directly compared to *Magneuptychia nebulosa*. The first accurate locality data for *Magneuptychia nebulosa* is also provided. During our examination of *Magneuptychia* specimens, we found that the *Magneuptychia nebulosa* type label was misapplied. Consequently, a lectotype designation for *Magneuptychia nebulosa* is included in order to objectively establish the identity of this taxon and stabilize the nomenclature.

## Materials and methods

Morphology. Male and female genitalia were dissected using the methods of Peña and Lamas (2005), except the female genitalia were stained for 30–60 seconds in dilute chlorazol black before being stored in 100% glycerin. Genitalia and external morphological characters were studied using a stereomicroscope and photographed by Canon EOS 50D. The terminology for genital and abdominal structures follows Klots (1956), except for the term brachia and aedeagus, which follows Muschamp (1915) and Peña and Lamas (2005) respectively. Forewing length was measured from the base to the tip of the wing using Vernier calipers. Nomenclature for wing venation follows the Comstock-Needham system as described by Miller (1970), and nomenclature for the areas and elements of the wing pattern follows Peña and Lamas (2005) and Neild (1996). All examined specimens, including type specimens, were examined from the following collections:

AN Andrew Neild collection, London, UK

BMNH The Natural History Museum, London, UK

IVIC The Venezuelan Institute for Scientific Research, Miranda, Venezuela

MGCL McGuire Center for Lepidoptera and Biodiversity, Florida Museum of Natural History, Florida, USA

Photographs of additional specimens (Warren et al. 2014) were also examined, as well as one dissection prepared at the Museo Entomológico Francisco Luis Gallego (MEFLG), Universidad Nacional de Colombia, Medellín, Colombia.

### Primary type specimen data

**Figure 1.**
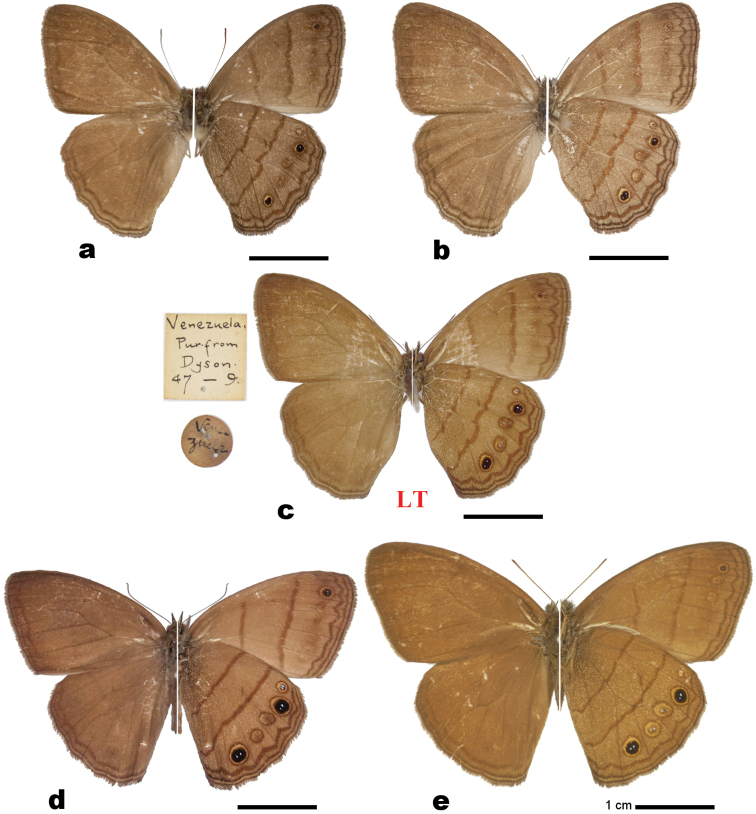
*Magneuptychia* adults (dorsal view on left, ventral view on right): **a** Male *Magneuptychia nebulosa* from Miranda, Venezuela **b** Female *Magneuptychia nebulosa* from Miranda, Venezuela **c** Lectotype of *Magneuptychia nebulosa* (photo credit: Trustees of the Natural History Museum, London) **d** Syntype of *Magneuptychia alcinoe* (photo credit: Trustees of the Natural History Museum, London) **e** Syntype of *Magneuptychia mimas* (photo credit: Trustees of the Natural History Museum, London).

*Magneuptychia nebulosa*: LECTOTYPE: Male (Fig. 1c): /*Venezuela/Venezuela Pur. from Dyson 47-9*/ (BMNH)

*Magneuptychia mimas*: SYNTYPE: Male (Fig. 1d): Type H.T./M#/Type of Species./Coroico. 6500ft., Bolivia. Garlepp./B.M. TYPE No. Rh3225. *Euptychia mimas*, Godm./BMNH(E) #983007/B.M.(N.H) Rhopalocera Slide No. 16843./T.G.H. 1953. 16./ Godman-Salvin Coll. 1904.-1. *Euptychia mimas*, Godm./ (BMNH)

*Magneuptychia alcinoe*: SYNTYPE: Female (Fig. 1e): Type/FELDER COLLN./Alcinoe /Rothschild Bequest B.M. 1939-1./Type of N. alcinoe Feld? = E. benedicta, Butl. of w it may be a good local form Comp w type E. benedicta Butler/ ECUADOR, Sarayacu. C. Buckley B.M. Type No. Rh3224. (BMNH)

### Genitalic dissections for *Magneuptychia alcinoe*

*Magneuptychia alcinoe*: Male, Ecuador: Tunguruhua Prov., Rio Machay, 1700m July 4–5 1993 J. P. W. Hall & K. R. Willmott (MGCL) KW-13-05; Male, Ecuador: Pichincha Prov., Rio Toachi, Union del Toachi 800m June 30 1993 J. P. W. Hall & K. R. Willmott (MGCL) KW-13-06; Colombia: Antioquia, Municipio de Amalfi, bosque Picardia N6°47'33", W75°06'36", 1050msnm October 12 2007 09:30:00 borde de bosque A. M. Velez (MEFLG); Male, Ecuador: Tandapi, Rio Pilaton, Pichincha 1500m August 10 1993 J. P. W. Hall & K. R. Willmott (MGCL) SN-14-57; Female, Ecuador: Esmeraldas Prov. Rd. Lita-Alto Tambo km.16. 850m June 6 1994 J. P. W. Hall & K. R. Willmott (MGCL) SN-14-60; Female, Colombia: Valle, Bitaco, 1700m Jan 7 1985 J. B. Sullivan (MGCL) SN-14-107.

## Results

### Magneuptychia nebulosa

Taxon classificationAnimaliaLepidopteraNymphalidae

(Butler, 1867)

Figs 1 , 2 and 4

Euptychia nebulosa Butler (1867: 479)

#### Redescription.

MALE: forewing length 19.6–21.5 mm (n=4).

**Wing shape.** Forewing with costa slightly convex, inner margin straight, outer margin almost straight, medium section slightly concave, anterior slightly convex, angular. Hind wing rounded, slightly angular, base of costa convex, inner margin convex beyond vein 3A, tornus rounded, outer margin slightly undulating, apex slightly angular.

**Wing venation.** Forewing recurrent vein absent; hindwing humeral vein present.

**Dorsal surface.** Forewing ground color brown, submarginal band dark brown, undulating, extending from apex towards tornus, delimiting the slightly darker area, marginal band dark brown, extending from apex towards tornus, fringe greyish brown.

Hindwing color brown, submarginal band dark brown, undulating, extending from apex towards tornus, convex in each cell; marginal band dark brown extending from apex towards tornus; postmarginal and tornal areas pale ocher, fringe greyish brown.

**Ventral surface.** Forewing ground color chestnut brown, paler along inner margin; discal band thin, straight, reddish brown, extending from radial vein to just beyond vein 2A; postdiscal band reddish brown, weakly undulating, slightly wider than discal band, extending from radial vein and traversing towards inner margin until vein 2A, curved basally in cell Cu2-2A, approximately 2/3 distance from wing base to apex; faint band between postdiscal and submarginal bands dark brown, broad, extending from radial vein to just beyond vein Cu2; submarginal band dark brown, undulating, extending from apex to tornus, becoming less undulating towards the tornus, parallel to postdiscal band; marginal band dark brown, darker than submarginal band, almost straight, extending from apex towards tornus; narrow band distal to marginal band, dark brown, traversing outer margin, delimiting remaining area and fringe; submarginal ocellus in cell M1-M2 black with two white pupils and orange ring; fringe brown.

Hindwing ground color same as forewing, overlaid with subtle whitish pearly cast along inner margin and basal area; discal band reddish brown, slightly distally curved, extending from costal margin to inner margin, approximately 1/3 distance from wing base to apex; undulating postdiscal band color and width same as discal band, weakly undulating, traversing from costal margin towards inner margin, slightly bent basally in discal cell, curved distally in cell Cu1-Cu2 and curved inwards towards the anal margin below vein 2A, approximately 2/3 distance from wing base to apex; submarginal band dark brown, extending from apex towards tornus, curved basally in each cell; dark brown marginal band traversing along marginal line from apex towards tornus; narrow band distal to marginal band, band dark brown, traversing along outer margin, delimiting remaining area and fringe; five submarginal ocelli present, cells Rs-M1 and M1-M2 each with black, orange-ringed ocellus with two white pupils, M1-M2 ocellus relatively large (compared to ocellus in cell Rs-M1), cells M2-M3 and M3-Cu1 each with orangish relatively small ocellus, sometimes indistinct, Cu1-Cu2 with black, orange-ringed ocellus with two white pupils, similar in size to M1-M2 ocellus; fringe greyish brown.

**Figure 2.**
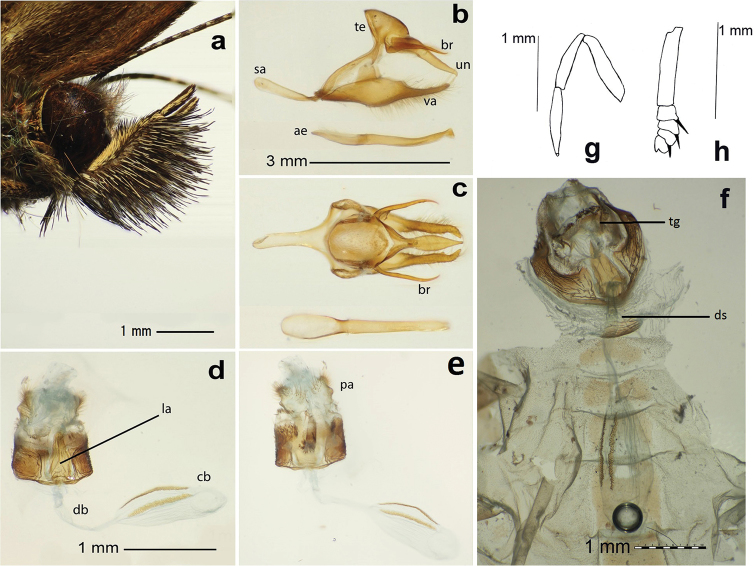
*Magneuptychia nebulosa*: **a** head in lateral view **b** male genitalia (SN-14-35) in lateral view **c** male genitalia in dorsal view **d** female genitalia (SN-14-59) in ventral view **e** female genitalia (SN-14-59) in dorsal view **f** female genitalia (SN-15-44) in dorsal view including sternites **g** male foreleg (tarsus, tibia and femur) **h** female foretarsus. Abbreviations: **ae**, aedeagus; **br**, brachia; **cb**, corpus bursae; **db**, ductus bursae; **la**, lamella antivaginalis; **pa**, papillae analis; **sa**, saccus; **ds**, ductus seminalis; **te**, tegumen; **tg**, tergite; **un**, uncus.

**Head.** (Fig. 2a) Eyes entirely brown, sparsely hairy; frons golden-brown, with whitish scales at base; labial palpi approximately 4 mm long, covered with brown and white scales, 2^nd^ segment densely covered in long black and white hairs ventrally, about 3-4 times as long as segment width, 3^rd^ segment about 40 % of 2^nd^ segment in length; antennae 8 mm long, approximately 40% of forewing length, scape and pedicel white, flagellum reddish brown dorsally, grey ventrally.

**Legs.** Tarsal segments reduced, foretarsus and tibia equal in length, femur slightly longer (Fig. 2g); midleg and hindleg covered with cream greyish scales, tarsus and tibia adorned with spines ventrally, a pair of tibial spurs present at distal end of tibia.

**Abdomen.** Eighth tergite and sternite reduced.

**Genitalia.** (Fig. 2b, c) Uncus elongate, slightly curved downward, with hooked apex, appears nearly elliptical in dorsal view, brachia hooked, pointed upward, positioned at approximately 45° angle, curved dorsally at middle of dorsal posterior margin, about 2/3 length of uncus; tegumen expanded dorsally, flattened ventrally; appendices angulares present; vinculum fused to posterior margin of tegumen, divided medially; saccus narrow and evenly wide in lateral view; costal margin of valvae slightly broadened medially, apex narrow, slightly curved towards uncus; aedeagus straight, broadening anteriorly.

FEMALE: Similar to male except as follows: Wings wider and rounder; forewing length 19.6–21.0 mm (n=8); dorsal ground color slightly paler; ventral ground color pale ocher, discal and postdiscal bands orange-brown; foretarsus divided into 5 segments (Fig. 2h); weakly sclerotized region between 7^th^ and 8^th^ sternite present in intersegmental membrane. **Female genitalia.** (Fig. 2d–f) Eighth tergite sclerotized, dorso-posterior area apparently weakly sclerotized; lamella antevaginalis sclerotized, sub-triangular in ventral view; 8^th^ segment heavily sclerotized laterally; ductus bursae unsclerotized, origin of ductus seminalis located approximately one third distance from ostium bursae to corpus bursae, corpus bursae equally long as ductus bursae, with two brownish signa.

#### Distribution.

**Figure 3.**
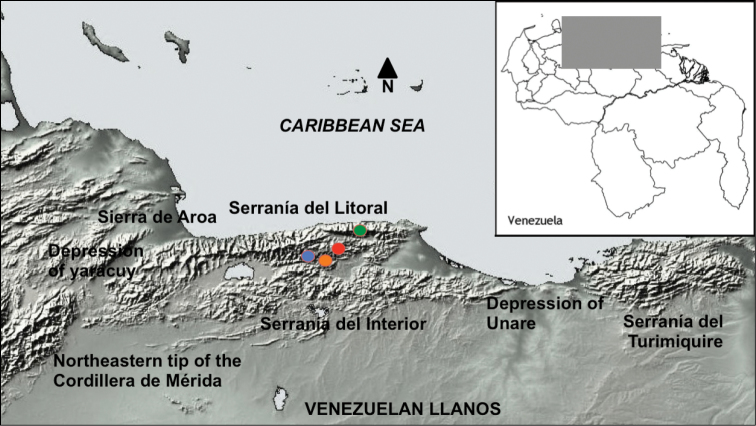
Map showing known localities for *Magneuptychia nebulosa*: blue dot = Quebrada Honda, El Jarillo; orange dot = Cerro Azul, Los Teques; red dot = Altos Pipe; green dot = Massif du Naiguata.

**Figure 4.**
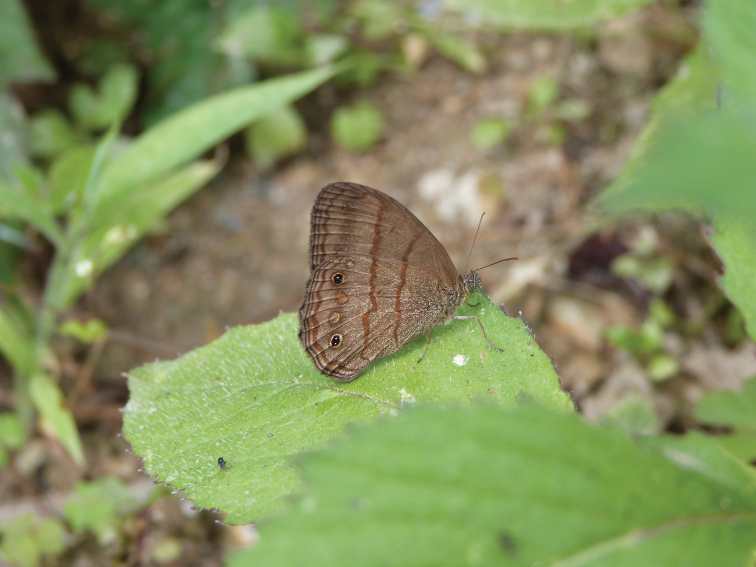
*Magneuptychia nebulosa* photographed in Quebrada Honda (photo by Cristóbal Ríos).

(Fig. 3) All known specimens of *Magneuptychia nebulosa* are from the slope of Serranía del Litoral in the Cordillera de la Costa: A huge mountainous district in northern Venezuela. However, one male specimen in the BMNH has a label that says ‘Colum’, implying it may have actually been collected in Columbia. On the other hand, it may be a misinterpretation of ‘Colonia [Tovar]’ (popular collecting site near Caracas) rewritten from an original label by BMNH staff. This label also says ‘Dys’, indicating the specimen was collected by Dyson, who had a lot of northern Venezuelan specimens and may have accidentally attached a Venezuelan label to this specimen (A. Neild, pers. comm.). A valid record from Colombia is needed to confirm its occurrence, as it is possible that this is a mislabeled Venezuelan specimen. The specimens in the BMNH bear no locality information other than the country of collection. However, *Magneuptychia nebulosa* specimens found in other collections have more accurate locality data: 4 males, VENEZUELA, Miranda, Altos de Pipe, 24 March 1973. J.B. Sulivan (MGCL) (3 dissection vials prepared: SN-14-33; SN-14-34; SN-14-35; 1 without label); 4 females, same data as males; Female, Venezuela: Miranda, Altos de Pipe J.B. Sullivan (MGCL) (1 dissection vial prepared: SN-14-59); 1 female, VENEZUELA, Miranda, Altos de Pipe, 17 March 1973. J.B. Sullivan (MGCL); 1 female, VENEZUELA, Miranda, Altos de Pipe, 24 July 1979. J.B. Sullivan (MGCL) (dissection vial without label); 1 female, VENEZUELA, Miranda, Altos de Pipe (IVIC site), above km11 turn off to Caracas to Los Teques rd, 1550-1650m, 13 Oct 2002 (AN). 1 female, VENEZUELA, Miranda, Cumbre Azul, 2km. NW of Los Teques, 11-0-1200m, 23.vii 1981 Lee D Miller (MGCL). 1 female, VENEZUELA, Dist. Federal Massif du Naiguta, 3 July 1957. R. Lichy (MGCL) (1 dissection vial prepared: SN-14-106); 1 female, VENEZUELA, Dist. Federal Massif du Naiguta, 1 September 1948. R. Lichy (MGCL) (dissection vial prepared: SN-15-44). In addition, the third author has recorded *Magneuptychia nebulosa* many times from Quebrada Honda (Fig. 4), El Jarillo (Miranda, Venezuela) and Altos de Pipe, see Suppl. material 1 for these data.

The female specimen in the AN collection is from a humid lower montane forest isolated on a ridge line along the southern slope of Cordillera de la Costa. The vegetation here has trees with a canopy over 15 m high, such as *Miconia* sp. (Melastomataceae), *Palicourea* sp. (Rubiaceae), *Clusia* sp. (Clusiaceae), and *Chusquea* sp. (Poaceae) (pers. obs.). Several true cloud forest inhabitants (e.g. *Evenus coronata* (Hewitson, 1865) (Lycaenidae), *Corades enyo enyo* (Hewitson, 1849) (Nymphalidae) and *Epiphile epicaste epicaste* (Hewitson, 1857) (Nymphalidae) are also recorded here (pers. obs.). Thus, it is reasonable to expect that *Magneuptychia nebulosa* can also be found in lower cloud forests on the slopes of the Cordillera de la Costa.

Godman and Salvin (1880) reported a record of *Magneuptychia nebulosa* from Chiriquí, Panama. However, his illustration is morphologically different from the lectotype, suggesting that this record is invalid. The ventral hindwing postdiscal band is not wavy as in *Magneuptychia nebulosa*. Three of the ocelli differ in both size and color, and the large apical ocellus has one pupil instead of two. The ventral forewing submarginal area lacks an obvious brown undulating band, and the ventral forewing discal band is much more curved. Forster (1964: p.104, figure 105, as *Yphthimoides nebulosa*) figured a male genitalia of *Magneuptychia nebulosa* based on a specimen from Bolivia. Although this genitalia appears to resemble those of *Magneuptychia nebulosa*, to judge from the curved ventral margin of tegumen and the developed cucullus, we believe this is not *Magneuptychia nebulosa* and this Bolivian record is invalid.

#### Diagnosis.

Phenotypically, *Magneuptychia nebulosa* most closely resembles *Magneuptychia alcinoe* and *Magneuptychia mimas*. These species can be distinguished from *Magneuptychia nebulosa* by size and wing pattern. *Magneuptychia nebulosa* is relatively small and possesses a wavy, somewhat irregular postdiscal band (slightly bent basally in discal cell, curved distally in cell Cu1-Cu2) of the ventral hind wing, whereas *Magneuptychia alcinoe* and *Magneuptychia mimas* are larger and have a straight hindwing postdiscal band. This straight ventral hindwing postdiscal band is also present in all other *Magneuptychia*. However, some of the members of *Paryphthimoides* (e.g. *Paryphthimoides poltys* (Prittwitz, 1865)) also exhibits this curved postdiscal band. *Magneuptychia nebulosa* possesses a rather reddish discal and post discal bands. The number of white pupils in the five ventral hindwing subapical ocelli varies within *Magneuptychia alcinoe* and is thus occasionally diagnostic; some specimens of *Magneuptychia alcinoe* have only one pupil in one of the ocellus (K. Willmott, pers. comm.), whereas *Magneuptychia nebulosa* always have two pupils in four ocelli, and one pupil in the larger, fifth ocellus. In addition, *Magneuptychia nebulosa* may be confused with a variation of *Magneuptychia modesta* (Butler, 1867), which is a species that seems to be very variable and is perhaps a complex of several species. However, *Magneuptychia nebulosa* differs from this taxon by the combination of the undulating ventral hindwing postdiscal band and double-pupilled ocelli in ventral hindwing cell M1-M2 (usually one in *Magneuptychia modesta*).

### Lectotype designation for *Magneuptychia nebulosa* (Butler, 1867)

*Magneuptychia nebulosa* was described from Venezuelan specimens. The type series was originally deposited in the Dyson collection and subsequently purchased by the BMNH in 1847 (G. Lamas, pers. comm.), where it is now deposited. The extant type series consists of one male (Fig. 1c) with two labels (/Venezuela/Venezuela Pur. from Dyson 47-9/) and one female that is currently labeled as the type (/Type/Venezuela/Venezuela Pur. from Dyson 47-9/Type/ B.M. Type No. Rh3223. Euptychia nebulosa Butl/). However, Butler’s description omits both the sex and the number of specimens examined, therefore, any “type” specimens ought to be syntypes. Because of the similarity of so many *Magneuptychia* species, it is important to select a lectotype to fix the name.

Butler provides a precise forewing measurement of 1.55 inches (39.37 mm). This theoretically makes it possible to deduce which specimen was the subject of Butler’s description, though the male and female syntypes have nearly identical forewing lengths of 39 mm and 40 mm, respectively. Butler’s description also clearly refers to five ocelli, three of which are relatively small with two pupils. Male ocelli have two pupils, but female ocelli only have one. Therefore, we designate the male specimen as the lectotype of *Magneuptychia nebulosa*. This is important because: (a) this male specimen most closely agrees with the original description, (b) it is in better condition than the female specimen, and (c) the male genitalia of euptychiine species are better known and are more commonly figured than female genitalia, and therefore have more scope to delimit species. The specific epithet *nebulosa* has been incorrectly applied to different taxa in Forster (1964) and D’Abrera (1988), as well as in the BMNH and other public collections (pers. obs.). This lectotype designation will remove doubt about the true identity of *Magneuptychia nebulosa*. The female specimen is consequently designated as a paralectotype. Note that this lectotype and paralectotype have a slightly different wing coloration probably because it faded over time.

### List of selected citations for *Magneuptychia nebulosa* being misapplied

Godman and Salvin (1880): p.86 (text); pl. 8 (ventral surface)

Weymer (1911): p.209 (text); pl. 48 (ventral surface)

Forster (1964): p.104 (male genitalia illustration, as *Yphthimoides nebulosa*)

Brown and Mielke (1967): p.91 (as *Yphthimoides nebulosa*)

D’Abrera (1988): p.776 (male dorsal and ventral surface)

## Discussion

The male and female genitalia of *Magneuptychia nebulosa* are extremely similar to those of *Magneuptychia alcinoe*. Despite dissecting several specimens per species, we could not find any convincing species-level differences except for their overall size, which appears to correlate to the differences in overall body size. However, the male genitalia exhibit some variation of the costal region and cucullus of the valvae, as well as variation in patterns of the cornuti. Further examination of these structures could provide diagnostic characters for male specimens of these taxa.

In general, most euptychiine species are distinguishable from their congeners by characters of the male and female genitalia, so the genitalic similarity would ordinarily suggest that *Magneuptychia nebulosa* and *Magneuptychia alcinoe* are conspecific. However, the small adult size of *Magneuptychia nebulosa*, its rather reddish bands, its curved ventral hindwing post discal band, and its rather small ocelli are all consistent and appear to be reliable characters to distinguish it from *Magneuptychia alcinoe*. We were not able to find records or specimens of *Magneuptychia alcinoe* from Cordillera de la Costa, nor *Magneuptychia nebulosa* from an area inhabited by *Magneuptychia alcinoe*, suggesting that the two species are allopatric. Cordillera de la Costa is isolated from the adjacent Sierra de Turimiquire and Cordillera de la Mérida by flat and scrubby lowlands, namely the depression of Yaracuy and the depression of Unare, respectively (see Fig. 3). However, we believe the evidence to support conspecificity (similar genitalia, no known area of sympatry) is weaker than the evidence supporting treatment as two different species (adult size, wing pattern, geographic isolation reinforced by specialized habitat preference). Therefore, we would settle the matter in favor of two species, and therefore treat *Magneuptychia nebulosa* as a valid species, presumably close to *Magneuptychia alcinoe*. Since *Magneuptychia nebulosa* was originally described as a species and never treated as a synonym, this treatment maintains the status quo. Although some might argue that it is not a reasonable decision to treat a Neotropical butterfly taxon known only from the Cordillera de la Costa as a valid species, we have two similar examples of montane cloud forest nymphalid species, *Memphis maria* Pycrz & Neild, 1996 and *Diaethria panthalis* (Honrath, 1884), which are also currently also known only from this mountain range (Neild 1996). On the other hand, it is true that there are many end-of-distribution-subspecies known from Cordillera de la Costa (e.g. *Pedaliodes manis ivica* Viloria & Pycrz, 2010), indicating that this kind of judgment is somewhat subjective.

Although the status of *Magneuptychia nebulosa* is currently resolved, other uncertainties about *Magneuptychia* remain. For example, the type of *Magneuptychia mimas* closely resembles that of *Magneuptychia alcinoe*, leading some to suggest that these taxa are conspecific, with the former merely being a Bolivian population of the latter. Conversely, these similarities may instead suggest the need for an “*alcinoe*” species group to distinguish these very similar taxa from other *Magneuptychia*. The relationships between *Magneuptychia nebulosa* and its congeners are still not fully understood; a revision of the genus is crucial to facilitate identification of euptychiine species in museum collections. Once we have a better understanding of Euptychiina and can reliably identify them, they can be used in broader, higher-impact studies of conservation and biogeography.

## Supplementary Material

XML Treatment for Magneuptychia nebulosa

## Supplementary materials

Supplementary material 1 Records for *Magneuptychia nebulosa* from Quebrada Honda, El Jarillo, Miranda, Venezuela Data type: Adobe PDF file Explanation note: Records for *Magneuptychia nebulosa* from Quebrada Honda, El Jarillo, Miranda, Venezuela; Altos de Pipe, Instituto venezolano de Investigaciones Científicas, Miranda, Venezuela: Cristóbal Ríos Málaver Leg: These following specimens are deposited in the reference collection of the Venezuelan Institute of Scientific Research IVIC, Altos de Pipe, Miranda, Venezuela. This dataset is made available under the Open Database License (http://opendatacommons.org/licenses/odbl/1.0/). The Open Database License (ODbL) is a license agreement intended to allow users to freely share, modify, and use this Dataset while maintaining this same freedom for others, provided that the original source and author(s) are credited.Shinichi Nakahara, Mario Alejandro Marín, Cristóbal Ríos-Málaver
