# Supplementary material for: Taxonomic status and redescription of Magneuptychia nebulosa (Butler, 1867) (Lepidoptera, Nymphalidae, Satyrinae) with a lectotype designation
Source: Zookeys. 2015 May 14;(503):135–47. doi: 10.3897/zookeys.503.9156 (PMC4440274; doi:10.3897/zookeys.503.9156)
Supplement: Supplementary material 1 — Records for Magneuptychia nebulosa from Quebrada Honda, El Jarillo, Miranda, Venezuela [file zookeys-503-135-s001.pdf]

Supplementary file1

Records for *M. nebulosa* from Quebrada Honda, El Jarillo, Estado Miranda Venezuela; Altos de Pipe, Instituto venezolano de Investigaciones Científicas, Estado Miranda Venezuela: Cristóbal Ríos Málaver Leg: These following specimens are deposited in the reference collection of the Venezuelan Institute of Scientific Research IVIC, Altos de Pipe, Miranda Venezuela.

18 males, VENEZUELA, Miranda, Quebrada Honda, el Jarillo, 1300 m, 6 March 2013, C. Ríos-Málaver; 6 females same data as males

2 males, VENEZUELA, Miranda, Quebrada Honda, el Jarillo, 1300 m, 3 April 2013, C. Ríos-Málaver; 1 female same data as males

3 males, VENEZUELA, Miranda, Quebrada Honda, el Jarillo, 1300 m, 30 May 2013, C. Ríos-Málaver

4 males, VENEZUELA, Miranda, Quebrada Honda, el Jarillo, 1300 m, 22 July 2013, C. Ríos-Málaver; 3 females same data as males

1 male, VENEZUELA, Miranda, Quebrada Honda, el Jarillo, 1300 m, 26 August 2013, C. Ríos-Málaver; 2 females same data as males

3 males, VENEZUELA, Miranda, Quebrada Honda, el Jarillo, 1300 m, 23 July 2013, C. Ríos-Málaver; 3 females same data as males

1 male, VENEZUELA, Miranda, Quebrada Honda, el Jarillo, 1300 m, 14 September 2013, C. Ríos-Málaver

1 male, VENEZUELA, Miranda, Quebrada Honda, el Jarillo, 1300 m, 15 September 2013, C. Ríos-Málaver

2 females, VENEZUELA, Miranda, Quebrada Honda, el Jarillo, 1300 m, 30 October 2013, C. Ríos-Málaver

2 females, VENEZUELA, Miranda, Quebrada Honda, el Jarillo, 1300 m, 30 October 2013, C. Ríos-Málaver

1 females, VENEZUELA, Miranda, Quebrada Honda, el Jarillo, 1300 m, 31 October 2013, C. Ríos-Málaver

3 males, VENEZUELA, Miranda, Altos de Pipe, 24 June 2012, C. Ríos-Málaver; 3 females same data as males

1 male, VENEZUELA, Miranda, Altos de Pipe, 29 June 2012, C. Ríos-Málaver; 2 females same data as males

1 female, VENEZUELA, Altos de Pipe, 30 June 2012, C. Ríos-Málaver

2 females, VENEZUELA, Altos de Pipe, 30 June 2011, C. Ríos-Málaver

1 female, VENEZUELA, Altos de Pipe, 30 July 2011, C. Ríos-Málaver

2 males, VENEZUELA, Altos de Pipe, 30 July 2012, C, Ríos-Málaver; 1 female same data as males

1 male, VENEZUELA, Altos de Pipe, 31 July 2012, C, Ríos-Málaver

1 male, VENEZUELA, Altos de Pipe, 23 August 2012, C, Ríos-Málaver

3 males, VENEZUELA, Altos de Pipe, 24 August 2012, C, Ríos-Málaver; 5 females same data as males

1 male, VENEZUELA, Altos de Pipe, 25 August 2012, C, Ríos-Málaver

1 male, VENEZUELA, Altos de Pipe, 31 August 2012, C, Ríos-Málaver

1 male, VENEZUELA, Altos de Pipe, 11 September 2012, C, Ríos-Málaver

2 males, VENEZUELA, Altos de Pipe, 16 September 2012, C, Ríos-Málaver

3 males, VENEZUELA, Altos de Pipe, 17 September 2012, C, Ríos-Málaver

2 males, VENEZUELA, Altos de Pipe, 29 September 2012, C, Ríos-Málaver; 1 female same data as males

1 male, VENEZUELA, Altos de Pipe, 15 October 2012, C, Ríos-Málaver

1 male, VENEZUELA, Altos de Pipe, 17 October 2012, C, Ríos-Málaver

1 male, VENEZUELA, Altos de Pipe, 19 November 2012, C, Ríos-Málaver

3males, VENEZUELA, Altos de Pipe, 21 November 2012, C, Ríos-Málaver

2 males, VENEZUELA, Altos de Pipe, 22 November 2012, C, Ríos-Málaver

5 males, VENEZUELA, Altos de Pipe, 23 November 2012, C, Ríos-Málaver; 2 females same data as males

2 males, VENEZUELA, Altos de Pipe, 24 November 2012, C, Ríos-Málaver

2 males, VENEZUELA, Altos de Pipe, 6 December 2012, C, Ríos-Málaver

1 male, VENEZUELA, Altos de Pipe, 7 December 2012, C, Ríos-Málaver

1 males, VENEZUELA, Altos de Pipe,8 December 2012, C, Ríos-Málaver

1 male, VENEZUELA, Altos de Pipe, 15 January 2013, C, Ríos-Málaver; 1 female same data as males

1 male, VENEZUELA, Altos de Pipe, 16 January 2013, C, Ríos-Málaver; 1 female same data as males

2 male, VENEZUELA, Altos de Pipe, 17 January 2013, C, Ríos-Málaver

2 male, VENEZUELA, Altos de Pipe, 9 February 2013, C, Ríos-Málaver

2 male, VENEZUELA, Altos de Pipe, 10 February 2013, C, Ríos-Málaver

1 male, VENEZUELA, Altos de Pipe, 16 February 2013, C, Ríos-Málaver; 2 females same data as males

2 male, VENEZUELA, Altos de Pipe, 17 February 2013, C, Ríos-Málaver

2 male, VENEZUELA, Altos de Pipe, 17 April 2013, C, Ríos-Málaver
